# The Importance of Culture in Addressing Domestic Violence for First Nation's Women

**DOI:** 10.3389/fpsyg.2018.00872

**Published:** 2018-06-04

**Authors:** Donna M. Klingspohn

**Affiliations:** Mental Health Recovery and Social Inclusion, School of Nursing and So Work, University of Hertfordshire, Hatfield, United Kingdom

**Keywords:** domestic violence, cultural safety, impacts of colonization and forced assimilation, intergenerational trauma, culturally specific vs. mainstream approaches to healing

## Abstract

Indigenous women in Canada face a range of health and social issues including domestic violence. Indigenous women (First Nations, Inuit and Métis) are six times more likely to be killed than non-Aboriginal women (Homicide in Canada, 2014; Miladinovic and Mulligan, 2015). Aboriginal women are 2.5 times more likely to be victims of violence than non-Aboriginal women (Robertson, 2010). These and other statistics highlight a significant difference in the level of violence experienced by Indigenous women to that experienced by women in the mainstream population in Canada. The historical impacts of colonization and forced assimilation are viewed as the main social determinant of health for aboriginal people in Canada, as they led to intergenerational trauma, with communities struggling today against discrimination, stigma, poverty and social exclusion. Most disturbing and damaging are the outcomes of domestic violence, mental health and addiction issues (Prussing, 2014). First Nation's women who want to leave a violent situation have limited access to helping services, as most are located in large cities and towns, far from remote reserves where many of the women live. Services were originally designed by and for the mainstream population. First Nation's women who manage to access these programs often find staff with limited cultural competence and program supports that have little cultural safety or relevance for them. Indigenous culture is defined in various levels of legislation as having a set of specific rights based on their historical ties to a particular region, with cultural or historical distinctiveness from the mainstream and other populations (Indigenous Peoples at the UN, 2014). In Canada, indigenous cultural beliefs are closely tied to belief in a creator, ancestors and the natural world, influencing their spirituality and their political perspectives (Waldram et al., 2006). Cultural safety, a concept that emerged in the 1980's in New Zealand, is viewed as an environment that is spiritually, socially, emotionally and physically safe for people; where cultural identity is recognized and valued through shared respect, meaning, knowledge and the experience of learning together. This paper will explore current evidence-based literature to determine if there is empirical evidence to support program policies and practices that reflect culturally safe, competent and relevant domestic violence services to address the cultural needs of Indigenous women in Canada.

## Background

European colonial ideology, reflective of racism and sexism, was used to repress and control indigenous peoples in Canada, and worldwide (New Zealand, Australia, South America and Africa), resulting in significant impacts to their health and social wellbeing (Bourassa et al., 2004).

Originally a matriarchal society, First Nation's women in Canada were respected and honored for their spiritual and mental strength; wealth, power and inheritance were passed down through mothers.

European colonists enacted legislation reflecting their patriarchal perspective, where women were not viewed as *persons*; recognizing only indigenous men as leaders of their communities. The respected and honored role of community leader was lost to aboriginal women for centuries (Cornet, 2001).

The passing of legislation in Canada such as the *Indian Act of 1876*, coupled with forced assimilation policies such as the residential school program, caused immeasurable harm, particularly for women. It has been claimed that South Africa's policy of apartheid was actually based on Canada's *Indian Act* (Saul, 2010).

The *Indian Act* denied women the right to possess land and marital property, unless they were a widow. However, even a widow could not inherit her husband's property upon his death as everything, including the house, went to his children. The *Act* changed slightly in 1884, with an amendment that allowed men to will their estate to their wives, but a wife could only receive the estate if the government's Indian Agent determined she was of “good moral character.”

“Once the *Indian Act* was passed, the responsibilities of our men and women changed drastically. As a result of being confined to a reserve, our traditional men and women lost their responsibilities in using their strengths, either physically or mentally. Women were thought of as property by our *O: gwe ho:we* men who became acculturated into believing that they had to think like white men. The entitlement to status under the Indian Act itself enabled that to happen, wherein the male would gain status and his wife, and his children would gain his status.”

Beverley Jacobs “International Law/The Great Law of Peace” (Jacobs, 2000, p. 108).

Residential Schools were the prime example of forced assimilation policies of the colonial government, where children were forcibly removed from their homes and communities to attend church administered residential schools. The real goal of these schools was to erase the traditional family and culture from the children and assimilate them into European colonial culture (Bombay et al., 2014). Instead of receiving a supportive educational experience, the children were abused—physically, sexually and psychologically—leaving lasting scars that have impacted generations of indigenous people. The Manitoba Justice Institute (1999) stated that “Residential schools laid the foundation for the epidemic today of domestic abuse and violence against Aboriginal women and children.” Though assimilation policies were officially renounced with a formal apology by the Canadian Federal Government in 2008, new legislation and policy has been very slow to develop (Prime Minister Stephen Harper's Statement of Apology, 2008).

With the changes in economies over the years, men in Indigenous communities lost traditional jobs such as fur trading. With few economic roles available for replacement in remote communities, many men found their gender roles under attack, with more women becoming the economic providers. The resulting sense of social, cultural and economic insecurity has become a powerful factor in domestic violence (Douglas, 2013).

These historical impacts left First Nation's women living in poverty and socially excluded, facing multiple stigmas and experiencing domestic violence, compounded by intergenerational trauma. The residential school experience left many women with mental health issues including complex post-traumatic stress disorder, depression and substance misuse, as well as a suicide rate that is five times that of non-aboriginal people in Canada (Belanger, 2014).

## Literature reveals

Critical evaluation and synthesis of the research literature is important to improve evidence-based decision making for policy and practice, by identifying valid evidence, bias, knowledge gaps and helping to separate fact from lore.

The objective of this review was to examine the evidence-based literature regarding effectiveness of mainstream, women serving agencies in delivering culturally safe and competent recovery services for aboriginal women experiencing domestic violence and also, a mental illness.

The main characteristics of the literature review, following Cooper's Taxonomy, helped systematically assess the quality of the literature and provided a guiding framework for subsequent research (Sipe and Stallings, 1996).

The main focus of the search was on historical impacts, correlated to significant health and social issues for First Nation's women and how they are currently addressed in policy and practice in service agencies.

The goal of the literature review was to reveal any particular cultural interventions that promote recovery and social inclusion, inform policy and practice to improve cultural safety and competency in recovery services and most importantly, improve the quality of life for indigenous women.

The innovative approach to this review was to find participatory research studies that allowed us to “hear the voices” of Indigenous women, to gain their perspectives on how they view the issue of domestic violence and identify strategies they would find effective while supporting their cultural beliefs. The returns of the review were subject to a thematic content analysis and separated into the identified emerging themes (see Appendix I).

## Summary of findings

Campbell (2002) conducted a review of the research on mental and physical health sequelae of domestic violence, in a paper published in the Lancet, titled “Health consequences of intimate partner violence,” concluding that the significant relationship of domestic abuse and mental health outcomes should be of concern and interest to clinicians as well as researchers.

Ramon (2015) echoed similar concerns in her paper “Intersectionalities: Intimate Partner Domestic Violence and Mental Health Within the European Context” demonstrating that the issue of domestic violence, concurrent with mental health problems, is a global issue that would benefit from the use of a recovery approach, changing the perspective of “victim” to “survivor. Jaffer and Mobina (1992) chaired a task force on Family violence in British Columbia. Their report, “*Is Anyone Listening*?” revealed nearly 30% of women in Canada experienced violence of a physical or sexual nature at least once in their relationship.

Sinha and Mair (2013) stated that British Columbia had the highest reported rates of violence against women of any province in Canada, indicating that not much has changed since the earlier report in 1992. One has to wonder what contributes to these statistics in BC.

In the article “*Understanding the elevated risk of partner violence against Aboriginal women*,” Brownridge (2008) looked at data from two national surveys comparing violence against aboriginal women to violence experienced by non-aboriginal women. The comparison revealed that aboriginal women are 4 times more likely to be victimized and posit this statistic to intergenerational impacts of colonization and loss of culture.

Data from the Homicide Survey indicated that Aboriginal women were disproportionally represented as homicide victims, and similarly, victimization data indicate that Aboriginal women have higher rates of self-reported spousal and non-spousal violence (Homicide in Canada, 2014).

The Four Worlds Centre for Development Learning (2003) examined Aboriginal domestic violence in Canada, to map the nature and extent of the problem and uncover the facilitating factors of family, community, cultural, professional and governmental systems, to develop an intervention framework and strategies to reduce violence. Bopp et al. (2003) despite some progress, there are still gaps between the incidence of domestic violence in Indigenous communities and the capacity of those communities and agencies to systematically and effectively address the problem.

Given the increasing statistics of domestic and other abuse in First Nation's communities, coupled with significant mental health issues, it is evident that the current service provision is not addressing the contributing factors.

It is posited that a strong prevention framework, based in cultural strategies and recovery principles would help to decrease both the incidence of domestic abuse and the development of concurrent mental health issues. However, current services focus on crises intervention and stabilization, lacking dedicated funding for prevention, leaving women in a cycle of violence and despair, with little hope for recovery.

Cultural safety, a concept that emerged in the 1980's in New Zealand, is viewed as an environment that is spiritually, socially, emotionally and physically safe for people; where cultural identity is recognized and valued through shared respect, meaning, knowledge and the experience of learning together (Williams, 1999). Many Aboriginal people don't utilize mainstream health care services, not only due to their remote location but also due to a lack of trust. They experience stereotyping and racism, consequently view Western health care and other services as alienating and intimidating. In their paper examining First Nation's women's experience with mainstream health care, Browne and Fiske (2001) found Indigenous women were marginalized and disadvantaged by encounters of racism, discrimination and structural inequalities.

In 2010, Canada endorsed the *United Nations (UN) Declaration on The Rights of Indigenous Peoples*, with Article 22 of the Declaration stating that:

“States shall take measures, in conjunction with indigenous peoples, to ensure that indigenous women and children enjoy the full protection and guarantees against all forms of violence and discrimination.” (page 9)

The Canadian government's Status of Women Committee released a parliamentary report in 2011, examining the issue of violence against Aboriginal women. They made recommendation that culturally appropriate services are *vitally* important, as there is a significant difference in the way domestic violence is viewed in the mainstream compared to aboriginal communities (Robertson, 2010).

## Current approaches/recommendations

Mainstream services often reflect a feminist approach; women who are victims of violence are supported to leave their relationship, develop self-sufficiency and learn to take care of themselves and their children. First Nation's women believe they are married for life, therefore, do not envision an outcome where they leave their husbands. Their goal is to unite the family and put to rest the intergenerational trauma that has ruled their lives for centuries (Robertson, 2010).

In evaluating projects of the Aboriginal Healing Foundation, cultural safety was highlighted as critical to healing, and that relationships based on acceptance, trust and safety are the first steps in the healing process (Castellano and Archibald, 2007).

In the *2011 Community Guide to end Violence Against Aboriginal Women*, there were five principles identified as best practice for cultural safety:

- Protocols -respect for cultural forms of engagement
- Personal Knowledge -understanding one's own cultural identity and sharing information about oneself to create a sense of equity and trust
- Process -engaging in mutual learning and evaluating from the service recipient perspective
- Positive Purpose -ensuring the process yields the right outcome for the service recipient according to *their* values, preferences, and lifestyle
- Partnership -promoting collaborative practice
- (Ontario Native Women's Association (ONWA), 2011).

In the recovery framework “*Honoring Our Strengths*”, culture is understood as the “outward expression of spirit,” and revitalization of the spirit is a vital best practice to ensuring health and well-being among Indigenous people. Recognition of the importance of ceremony, language and traditions help to focus on strengths and reconnection with oneself, their history, family, community and land (Exhibition et al., 2010).

“Cultural safety extends beyond cultural awareness and sensitivity within services and includes reflecting on cultural, historical and structural differences and power relationships within the care that is provided. It involves a process of ongoing self-reflection and organizational growth for service providers, and the system as a whole, to respond effectively to First Nations”

(Honouring our Strengths, 2011, p. 8).

(The Indigenous Physicians Association of Canada the Royal College of Physicians Surgeons of Canada, 2009) believe that if mainstream health care is to be truly effective in improving the health of First Nations, Inuit, and Métis clients, it *must* provide culturally safe care. Any definition of cultural safety should include a strategic and pragmatic plan to change the way healthcare is delivered to Aboriginal people.

The traditional teaching of the Medicine Wheel guides a holistic healing process, examining the intersectionalities of domestic violence in relation to physical, mental, emotional and spiritual domains (Dapice, 2006). Many healers and indigenous services use the wheel's domains as the underpinning of their approach to achieving balance in well-being.

Culture is all about history and society; complex and dynamic, as opposed to a given set of beliefs or practices. Service providers must understand that the ongoing impacts of colonization have had a range of negative effects on First Nations peoples' health, rather than just having an understanding of specific cultural practices (Browne and Varcoe, 2006).

## Culture and trauma informed practice

The (First Nations Health Authority, 2016) in their Policy Statement on Cultural Safety and Humility, recommends that all health services:

“Increase opportunities to educate health care professionals, those training to become health professionals, and others working in the health system on the history of First Nations health, as well as the concepts of cultural safety, and cultural humility and the relevance to First Nations health. Training to include: Recognizing the role of history and society, their impacts, and their relationship to culture in shaping health and health experiences of First Nations. This includes recognizing the role of trauma and offering trauma-informed care” (p. 15).

Trauma informed care and practice embraces a recovery focused, strengths-based approach, with an understanding and response to the impacts of trauma, where psychological, physical and emotional safety are paramount (for providers and service users) and provides opportunities for control, empowerment and recovery.

Given the history of intergenerational trauma experienced by Indigenous women, all service providers delivering services to address domestic violence must have a clear understanding of the traumatic effects of colonization, its impacts on indigenous women and their culture and develop competency in the types of cultural approaches that will be effective.

## Role of prevention

A review of the literature related to cultural interventions for domestic violence reveals the need for primary, secondary and tertiary prevention approaches (Shea et al., 2010).

Kiyoshk (2003) promotes primary prevention to reduce risk factors for family violence by integrating spiritual practices and ceremonies into family group counseling, utilizing cultural healing methods such as smudging, the talking circle, and the sweat lodge with Aboriginal men.

In a study on secondary prevention to stop risk factors becoming actual violence, Norton and Manson (1997) reported on the effectiveness of home visits to conduct weekly family violence groups that incorporated sharing of food and talking circles. Building a positive relationship with the counselor improved the outcomes for women taking part in the family violence program.

Willmon-Haque and Bigfoot (2008) reviewed literature on historical trauma and poverty, calling for early intervention in family violence to prevent further trauma to children. Services such as advocacy, promoting cultural awareness and culturally relevant services as well as involving communities in research are seen as vital to supporting mental health.

A recent development in prevention focuses on the responsibilities of the men. A program called (The Moose Hide Campaign, 2016) begun as a grassroots organization in BC to bring awareness to violence against women and children, has now expanded across Canada. Men are invited to join and stand against violence with a commitment to “honor, respect, and protect the women and children in their lives and to work together to end violence against women and children.” Though the campaign began in the Indigenous community, non-indigenous men are welcomed, with the movement now having well over one million members (moosehide campaign.ca).

Battered Women's Support Services in Vancouver (BWSS, 2018) have successfully utilized youth targeted social media, to spread key prevention messages to stop violence against women and children (https://www.bwss.org/20-ways-youth-can-prevent-violence)^1^.

The Federal Government has a Family Violence Prevention Program designed to improve the safety of indigenous women, children and families. Funding is provided for prevention projects such as “awareness campaigns, conferences, workshops, stress and anger management seminars, support groups, and community needs assessments on and off reserve” (Government of Canada, Family Violence Prevention Program, 2017); (https://www.aadnc-aandc.gc.ca/eng/1100100035253/1100100035254).

Annualized funds also support a network of shelters across Canada with services for women and children living on reserve.

Tertiary prevention looks at reducing of the worst effects of domestic violence. Heilbron and Guttman (2000) examined the outcomes for women who participated in a Healing Circle, providing a spiritual framework for group therapy. The women reported feeling empowered, safe, and comfortable. The healing circle sharing of Indigenous traditions and teachings provided “personal meaning” for the women, helping them develop a stronger connection to Aboriginal healing methods and ultimately their own cultural roots.

## Conclusions

Reflecting on the literature related to domestic violence and Indigenous women, there are indications of a clear need for the recognition and inclusion of culture in any helping services. Aboriginal women face multiple forms of stigma; being an aboriginal female, having a mental health issue, and being a victim of domestic violence (Hurtado, 1997). Statistics related to domestic violence show a disturbing difference in the incidence of violence and death comparing statistics of indigenous women to women in the mainstream. The key difference between the two groups is based in historical impacts. Colonial legislation and forced assimilation policies, such as the residential school program, led to significant traumatic impacts to the health and wellbeing of Indigenous people, and in particular, for women for multiple generations.

Moore (2001) states any recovery strategies developed for First Nation's women must be self-determined, working holistically toward reducing the negative impacts of all contributing factors, such as social exclusion, hopelessness and poverty, on the well-being of these women. This collaborative recovery approach would help to change the perspective of the women from victim to survivor.

Domestic violence services developed for the mainstream embrace a feminist model that does not fully consider the unique cultural needs of Indigenous women, nor are indigenous cultural principles clearly reflected in the services currently provided. Staff working in such programs must be educated in the history and culture of Indigenous women and be able to offer trauma informed practice with cultural safety and competency (Douglas, 2013). It has been proposed that a restorative justice model, embraced by First Nation's communities, is culturally a better fit, however there are concerns that it may lack the accountability that is needed to address the seriousness of the abuse (Ptacek, 2009).

Upon consideration of all recommendations found in the current literature, there is overwhelming evidence that effective domestic violence services delivered to Indigenous women must actually involve the women in the planning and delivery of recovery services that embrace their traditional beliefs and cultural principles. Planning of policies and program services should also involve key persons in the community, including Elders and tribal band council members. A range of Indigenous healing strategies should be offered to women and their families, such as smudging, talking/healing circles and sweat lodges to help reconnect to their ancient culture and address the issue of intergenerational trauma. Prevention programs, targeted to men and youth, may be effective in changing attitudes and behaviors toward indigenous women and girls.

Effective programs follow a cultural path, help one regain balance and share in what is viewed as the “circle of life.” The use of healing traditions, such as those mentioned above, are designed to address the domains of the Medicine Wheel in planning holistic programs to reflect balance in the spiritual, emotional, mental and physical realms (Hunter et al., 2006). While we see some progress in developing culturally appropriate policy and programs, there is still a long way to go.

As a signatory in 2010 to the *United Nations (UN) Declaration on The Rights of Indigenous Peoples*, Canada must meet those declared obligations by developing effective services to end discrimination and violence toward Indigenous women and children (United Nations General Assembly, 2007).

A Message from the Elders:

“Women, like Mother Earth are life givers and nurturers of our children, families, communities and nations. By gathering our Indigenous women, we are stepping into our traditional values, ceremonies, teachings and cultures; embodying personal healing and connections to our ancestors, and to future generations to come. With the guidance of our elders, personal healing helps us to hold our connection to Mother Earth, the Creator and all our relations.”

Bev Gillard, Cree Elder Chairperson of the Elders Advisory Council, Circle of Indigenous Nations Society in the West Kootenay and Boundary region (Province of British Columbia, 2016).

## Search engines

A wide range of were utilized to review databases such as CINAHL; MEDLINE; Wiley InterScience; ATSIHealth; ProQuest; EBSCO; Google Scholar; Voyager; EMBASE; PubMed. As well, academic databases searched were: Women's Studies International; iPortal for Indigenous Studies (University of Saskatchewan); OISE's Aboriginal Peoples Curricula Database (University of Toronto); Indigenous Research Resources (Okanagan College); Indigenous Foundations (University of British Columbia).

## Author's note

DK has worked in the field of Psychiatric Nursing in Canada for over 35 years. Her master's dissertation in mental health recovery and social inclusion focused on the need to hear the voices of Indigenous women in conducting research and planning for services to address mental health and domestic violence issues. The issues of cultural safety and competency have guided her work in this area.

## Author Contributions

The author confirms being the sole contributor of this work and approved it for publication.

### Conflict of interest statement

The author declares that the research was conducted in the absence of any commercial or financial relationships that could be construed as a potential conflict of interest.

## Footnotes

^1^Battered Women's Support Services, *20 Ways Youth Can Prevent Violence Against Girls and Women*. https://www.bwss.org/20-ways-youth-can-prevent-violence/
