# Supplementary material for: The Importance of Culture in Addressing Domestic Violence for First Nation's Women
Source: Front Psychol. 2018 Jun 4;9:872. doi: 10.3389/fpsyg.2018.00872 (PMC5996937; doi:10.3389/fpsyg.2018.00872)
Supplement: Supplementary file 1 [file Data_Sheet_1.docx]

**Appendix I**

**Themes Emerging from a Review of the Literature**

Yellow – related to deficits in current services regarding to cultural and human rights needs

Green – issues of abuse concurrent with mental health

Aqua – Aboriginal women’s narrative and expressed needs

Grey – mainstream program deficits

Red - Residential school outcomes

No Colour – basic statistics and research guidelines

| **Article/Citation** | **Content/Issue** | **Comments/Conclusions** |
| --- | --- | --- |
|  |  |  |
| Ida, D.J., 2007. Cultural competency and recovery within diverse populations. *Psychiatric Rehabilitation Journal*, *31*(1), p.49. | Recovery must consider the role of culture and language, looking at the individual within the context of racism, sexism, colonization and poverty as well as the stigma and shame associated with having a mental illness | The current mental health system has **neglected to incorporate, respect or understand the histories, traditions, beliefs, languages and value systems** of culturally diverse groups. |
| Campbell, J.C., 2002. Health consequences of intimate partner violence. *The Lancet*, *359*(9314), pp.1331-1336. | Review of research on the mental and physical health sequelae of domestic violence | The **concurrent relations of abuse and mental-health outcomes should be of interest to practitioners as well as researchers** |
|  |  |  |
| Wathen, N., 2012. Health Impacts of Violent Victimization on Women and their Children. *Ottawa, ON: Department of Justice Canada. Retrieved November*, *7*, p.2013. | Growing evidence of the strong links between violence against women and significant physical and mental health impairment | Associated with high rates of **depression, anxiety disorders (especially post-traumatic stress disorder). For Aboriginal women with abuse histories, rates may be higher**, though they may rate health at the same levels as do non-Aboriginal women. |
|  |  |  |
| Adelson, N., 2005. The embodiment of inequity: Health disparities in Aboriginal Canada. *Canadian Journal of Public Health/Revue Canadienne de Sante'e Publique*, pp.S45-S61. | Lit review showing health disparities are directly and indirectly associated with social, economic, cultural and political inequities; the result is a disproportionate burden of ill health and social suffering for First Nations | **Research and policy must address the contemporary realities of Aboriginal health and well-being, including the individual and community-based effects of health disparities** and the direct and indirect sources of those disparities |
|  |  |  |
| Benoit, C., Carroll, D. and Chaudhry, M., 2003. In search of a healing place: Aboriginal women in Vancouver's downtown eastside. *Social science & medicine*, *56*(4), pp.821-833. | Aboriginal people face formidable barriers in accessing culturally appropriate and timely care. Research with Aboriginal women asked them whether the appropriate services and educational programs to address their health care needs were being provided in the inner city. | **Aboriginal women expressed a need for a Healing Place, where their health concerns are addressed in an integrated manner, where they are respected and given the opportunity to shape and influence decision-making** about services that impact their healing. |
|  |  |  |
| Browne, A.J. and Fiske, J.A., 2001. First Nations women’s encounters with mainstream health care services. *Western journal of nursing research*, *23*(2), pp.126-147. | Study examining **mainstream health care encounters** from the viewpoint of First Nations women from a remote reserve in British Columbia. Perspectives from the concept of cultural safety | **Encounters were shaped by racism, discrimination, and structural inequities that marginalize and disadvantage First Nations women**. Their experiences have historical, political, and economic significance and are reflective of wider postcolonial relations |
| Mann, J.M., Gostin, L., Gruskin, S., Brennan, T., Lazzarini, Z. and Fineberg, H.V., 1994. Health and human rights. *Health and human rights*, pp.6-23. | Provisional framework for exploring potential collaboration in health and human rights. The interdependence of health and human rights has substantial conceptual and practical implications affecting policies, programs and practice | **Health and human rights are complementary approaches for defining and advancing human well-being.** |
|  |  |  |
|  |  |  |
| Smye, V. and Mussell, B., 2001. Aboriginal mental health-What works best? Simon Fraser University, Summit repository | Discussion paper from Aboriginal Mental Health committee in BC due to **concerns that mental health service delivery, including the field of community psychiatry, did not adequately – or appropriately – deal with the needs of Aboriginal people**. | Mental health services and **programs must reformed**. Service priorities must be: Culturally relevant/ safe;  Strength-based; Integrated; Support for Mental Health Community-Based Initiatives; Accredited Education and Training; Research -Evidence-based practices |
| Alfred, T., 2009. Colonialism and state dependency. *Journal de la santé autochtone*, *5*, pp.42-60. | Review and consideration of the literature, identifying a direct relationship between government laws and policies applied to Indigenous peoples and the myriad mental and physical health problems and economic deprivations. | **Recommendations** for developing policy responses to the situation which are oriented towards **supporting and facilitating Indigenous people’s reconnection to their homelands, restoration of land-based cultural practices and the rebuilding of indigenous communities.** |
|  |  |  |
| Cochran, P.A., Marshall, C.A., Garcia-Downing, C., Kendall, E., Cook, D., McCubbin, L. and Gover, R.M.S., 2008. Indigenous ways of knowing: Implications for participatory research and community. *American Journal of Public Health*, *98*(1), p.22. | The way researchers acquire knowledge in indigenous communities may be as critical for eliminating health disparities as the actual knowledge that is gained about a particular health problem | Researchers working with indigenous communities must continue to **resolve conflict between the values of the academic setting and those of the community**. It is important to consider the ways of knowing that exist in indigenous communities when developing research methods. |
| Tait, C. L. (2013). Resituating the ethical gaze: government morality and the local worlds of impoverished Indigenous women. *International Journal of Circumpolar Health*, *72*, 10.3402/ijch.v72i0.21207. http://doi.org/10.3402/ijch.v72i0.21207 | **Life narrative of a First Nation’s women**. Over generations, government policies have impacted upon the lives of Indigenous peoples of Canada in unique and often devastating ways. In this context, Indigenous women who **struggle with poverty, mental illness, trauma** and substance abuse are among the most vulnerable. | Government policies and the practices they generate must be first and foremost **to ensure that individuals, families and groups are not left worse off than prior to a government policy** impacting upon their life |
|  |  |  |
|  |  |  |
| Stout, M., Kipling, G. and Stout, R., 2001. Aboriginal women’s health research. *Winnipeg: Canadian Women’s Health Network*. | Provides an overview of key health and health-related indicators for Aboriginal women in Canada with a **critical review and synthesis of research and other initiatives on Aboriginal women’s health and priority recommendations** | To promote the indigenization of the research process; to engage Aboriginal women in the research process; to address gaps and weaknesses in Aboriginal women’s health research, Work with **Aboriginal women’s health researchers to develop culturally-appropriate methodologies** |
|  |  |  |
|  |  |  |
|  |  |  |
| MacMillan, H.L., Jamieson, E., Walsh, C.A., Wong, M.Y.Y., Faries, E.J., McCue, H., MacMillan, A.B. and Offord, D.D.R., 2008. First Nations women’s mental health: results from an Ontario survey. *Archives of women's mental health*, *11*(2), pp.109-115. | The mental health of First Nations women living on reserve in Ontario and compares these findings with results from the National Population Health Survey (NPHS) | Compared with NPHS women, First Nations women reported **significantly higher rates of depression (18% vs 9%)** Given the burden of suffering associated with depression, it is important to examine risk and protective factors specific to First Nations women. |
|  |  |  |
|  |  |  |
| E.J. Peters 2006 "[W]e do not lose our treaty rights outside the reserve": challenging the scales of social service provision for First Nations women in Canadian cities  Geojournal, 65 (2006), pp. 315–327 | Canadian federal government takes responsibility for social services for First Nations people on reserves, with the provinces responsible for First Nations people in cities. This means First Nations women as individuals have Aboriginal rights only on reserves; off reserve women are seen as part of mainstream society in urban areas. | First Nations women have challenged the definitions of their identities embedded in these scales of service provision. In presenting alternative geographies for organizing the provision of services, they demonstrate the **importance of paying attention to the diversity of women’s everyday geographies in the city.** |
|  |  |  |
| Basile, S 2012 Guidelines for Research with Aboriginal Women, Université du Québec en Abitibi-Témiscamingue  (UQAT). Published by Quebec Native Women (QNW) | Guidelines prepared for Aboriginal women, decision-makers and managers in the Aboriginal communities, and researchers interested in examining issues related to Aboriginal women. | Suggestions: **Culturally relevant gender-based comparative analysis**; **Include women in initial contacts made before starting the research; Hold extensive consultations with the community; Involve Aboriginal women in the task of defining the research subject, choosing the methodology and preparing the materials (e.g., questionnaire), and in all other steps of the research; Base the study on local needs and priorities identified by the women themselves; Consider Aboriginal knowledge; Restore the voice of Aboriginal women; Uphold the basic values of respect, trust, knowledge, balance, fairness and decision-making power** throughout the entire research process; **Adopt an inclusive attitude and approach, and maintain an ongoing dialogue and** an effective and sincere partnership between the researchers and the Aboriginal women |
|  |  |  |
| Ethical Guidelines for Aboriginal Women’s Health Reasearch,2004 Saskatoon Aboriginal Women’s Health Research Committee, Prairie Women’s Health Centre of Excellence, Winnipeg, Manitoba | Guidelines to uphold standards of best practice in research with appropriate respect given to the cultures, languages, knowledge and values of Aboriginal peoples’ legitimate knowledge. | **Researchers shall conscientiously address themselves to the following questions**: Are there perspectives on the subject of inquiry that are distinctively Aboriginal; What Aboriginal sources are appropriate to shed light on those perspectives; Is proficiency in an Aboriginal language required to explore these perspectives and sources; Are there particular protocols or approaches required to access the relevant knowledge; Does Aboriginal knowledge challenge in any way assumptions brought to the subject from previous research; How will Aboriginal knowledge or perspectives portrayed in research produced be validated; How will all comments of participants be validated; How will comments be recorded to ensure the context of the participant is portrayed |
|  |  |  |
|  |  |  |
|  |  |  |
| Kirmayer, L., 2012. Rethinking cultural competence. *Transcultural Psychiatry*, *49*(2), p.149. | **There is wide recognition that health services and mental health promotion must consider culture to be ethically sound and clinically effective** (Anderson, Scrimshaw, Fullilove, Fielding, & Normand, 2003; Brach & Fraserirector, 2000). | Cultural competence has emerged as an **important counter-balance to the movement for evidence-based mental health care**, which tends to lead to a ‘‘one-size-fits-all’’ approach (Whitley, 2007) There is a **great need for research on the processes of implementation, clinical effectiveness, wider social impact and outcomes of culturally competent services and interventions.** |
|  |  |  |
| Sue, D.W., Arredondo, P. and McDavis, R.J., 1992. Multicultural counseling competencies and standards: A call to the profession. *Journal of Counseling and Development*, *70*(4), pp.477-486. | A body of literature exists that documents the widespread ineffectiveness of traditional counseling approaches and techniques when applied to racial and ethnic minority populations (Bernal & Padilla, 1982; Casas, 1982; Casas, Ponterotto, & Gutierrez, 1986; Ibrahim & Arredondo, 1986; President’s Commission on Mental Health, 1978; Smith, 1982; Sue, 1990; Sue & Sue, 1990; Sue et al., 1982). It is apparent that the major reason for therapeutic ineffectiveness lies in the training of mental health professionals (Sue, Akutsu, & Higashi, 1985). | A **culturally skilled counselor is one who is**: actively in the process of becoming aware of his or her own assumptions about human behavior, values, biases, preconceived notions, personal limitations, and so forth; actively attempts to understand the worldview of his or her culturally different client without negative judgments; in the process of actively developing and practicing appropriate, relevant, and sensitive intervention strategies and skills in working with his or her culturally different clients |
|  |  |  |
|  |  |  |
| Kirmayer, L., Simpson, C. and Cargo, M., 2003. Healing traditions: Culture, community and mental health promotion with Canadian Aboriginal peoples. *Australasian Psychiatry*, *11*(sup1), pp.S15-S23. | To **identify issues and concepts to guide the development of culturally appropriate mental health promotion strategies with Aboriginal populations** and communities in Canada. | There is **clear and compelling evidence that the long history of cultural oppression and marginalization has contributed to the high levels of mental health problems found in many communities**. There is evidence that **strengthening ethno-cultural identity, community integration and political empowerment can contribute to improving mental health** in this population. Research on variations in the prevalence of mental health disorders across communities may provide important information about community-level variables to supplement literature that focuses primarily on individual-level factors. |
|  |  |  |
|  |  |  |
| Fischbach, R.L. and Herbert, B., 1997. Domestic violence and mental health: correlates and conundrums within and across cultures. *Social science & medicine*, *45*(8), pp.1161-1176. | While still fragmentary, accruing data reveal strengthening associations between domestic violence and mental health.  Depression, stress-related syndromes, chemical dependency and substance (ab)use, and suicide are consequences observed in the context of violence in women's lives. | **Emerging social, legal, medical, and educational strategies, often culture specific, offer novel local models to promote social change beginning with raising the status of women**. Additional research is needed to promote the recognition, intervention, and prevention of domestic violence that are both locally specific and internationally instructuve. |
| Ramon, S., 2015. Intersectionalities: Intimate Partner Domestic Violence and Mental Health Within the European Context. *Intersectionalities: A Global Journal of Social Work Analysis, Research, Polity, and Practice*, *4*(2), pp.76-100. | Article highlights the traumatic impact of intimate partner domestic violence (IPDV) on women, the complexity of their responses to it, its impact on their identities, and their resulting social position | A case is put forward for **applying the new meaning of recovery in mental health to women experiencing IPDV. That approach has the potential to provide a positive contribution, enabling them to move from being victims to becoming survivors,** |
|  |  |  |
| Satzewich, V. and Wotherspoon, T., 2000. *First Nations: Race, class and gender relations* (Vol. 7). University of Regina Press. | Book - Despite the collection of massive amounts of data and existence of numerous studies on aboriginal people, there has been no improvement to the quality of living conditions for aboriginal people. Canadian sociologists have not kept pace with their counterparts in other countries | Provides an alternative perspective of aboriginal /non-aboriginal relations to spark further research and debate in these areas. |
|  |  |  |
|  |  |  |
| Brownridge, D.A., 2008. **Understanding the elevated risk of partner violence against Aboriginal women:** A comparison of two nationally representative surveys of Canada. *Journal of Family Violence*, *23*(5), pp.353-367. | Data from two nationwide surveys reviewed to look at the issue of violence against aboriginal women compared to non-aboriginal women; found to be 4 x the mainstream rate. | Results show **increased odds of victimization of aboriginal women is probably linked to impacts of colonization**. Further research is needed to provide direct evidence of a connection between cultural loss and aboriginal women’s elevated odds of violent victimization |
|  |  |  |
|  |  |  |
| Wilson, K., 2003. Therapeutic landscapes and First Nations peoples: an exploration of culture, health and place. *Health & place*, *9*(2), pp.83-93. | This paper contributes to an expanding body of research within Health Geography that focuses on the role of therapeutic landscapes in shaping health. Therapeutic landscapes demonstrate the importance of places for maintaining physical, emotional, mental and spiritual health. Meanings of place and the relationship between place and health have culturally specific dimensions, yet these tend to be overlooked especially with respect to First Nations peoples | This paper presents the results of 17 in-depth interviews conducted with Anishinabek (Ojibway and Odawa) living in one First Nations community in northern Ontario, Canada.  The findings from the interviews demonstrate that **culture is an important component of the link between health and place in everyday life.** Incorporating First Nations peoples’ perspectives of health and place reveals that the current conceptualizations of health and place within the Geography of Health literature are only partial. |
| Elias, B., Mignone, J., Hall, M., Hong, S.P., Hart, L. and Sareen, J., 2012. Trauma and suicide behaviour histories among a Canadian indigenous population: An empirical exploration of the potential role of Canada's **residential school system**. *Social Science & Medicine*, *74*(10), pp.1560-1569. | In Canada, qualitative evidence has suggested that the Indian Residential School System set in motion a cycle of trauma, with some survivors reporting subsequent abuse, suicide, and other related behaviours. It has been further postulated that the effects of trauma can also be passed inter-generationally. | For First Nations adults who did not attend a residential school, it was found that age **28–44, female sex, not having a partner, and having a parent or grandparent who attended a residential school was associated with a history of abuse**.  This is the **first study to empirically demonstrate, at the population level, the mental health impact of the residential school system on survivors and their children.** |
| Valaskakis, G.G., Stout, M.D. and Guimond, E., 2009. *Restoring the balance: First Nations women, community, and culture*. Univ. of Manitoba Press. | Book -The contributions of First Nations women to their culture and communities is missing from the discourse on self-determination. | Women are the guardians of indigenous traditions, practices and beliefs – thus are agents of change for the families and the nations. |
| Cargo, M. and Mercer, S.L., 2008. The value and challenges of **participatory research**: Strengthening its practice*. *Annu. Rev. Public Health*, *29*, pp.325-350. | A critical review of the empirical and non-empirical PR literature, re: use of participatory research (PR) approaches to address pressing public health issues reflects PR's potential for bridging gaps between research and practice, addressing social and environmental justice and enabling people to gain control over determinants of their health. | An integrative practice framework that features five essential domains and provides a **structured process for developing and maintaining PR partnerships, designing and implementing PR efforts, and evaluating the intermediate and long-term outcomes of descriptive, etiological, and intervention PR** studies Advances to the practice of PR over the next decade will require establishing the effectiveness of PR in achieving health outcomes and linking PR practices, processes, and core elements to health outcomes. |
| Minkler, M., 2005. Community-based research partnerships: challenges and opportunities. *Journal of Urban Health*, *82*, pp.ii3-ii12. | **Community-based participatory research (CBPR) is presented as a promising collaborative approach** that combines systematic inquiry, participation, and action to address urban health problems | The concepts of partnership synergy and cultural humility, together with protocols such as Green et al.’s guidelines for appraising CBPR projects, are highlighted as useful tools for urban health researchers seeking to apply this collaborative approach and to deal effectively with the difficult ethical challenges it can present. |
| Brunen, L., 2000. *Aboriginal women with addictions: A discussion paper on triple marginalization in the health care system*. Northern Secretariat of the BC Centre of Excellence for Women's Health. | Paper to define and develop a viable research question in women's health, identify and assess some relevant literature, and provide recommendations for further research. Synthesis of available material **explores racism in health care**; In particular the ways in which First Nations women who misuse substances are triply marginalized in the health care system, with devastating impact. | **Lit review determined that there is little direct material relating how racism and substance misuse interact for Aboriginal women accessing mainstream health care.** Health care system perpetuates the inherent values, beliefs, and attitudes of mainstream society. **Stigma towards mental health and substance misuse. Devaluing of women in general** |
|  |  |  |
| Four Worlds Centre for Development Learning, 2003. *Aboriginal domestic violence in Canada*. Ottawa, Ontario, Canada: Aboriginal Healing Foundation. | This study focuses on domestic violence and abuse in Aboriginal communities in Canada. to develop a generic map of the problem of Aboriginal domestic violence and abuse that simultaneously describes the full nature and extent of the problem and also uncovers the dynamics of family, community, cultural, professional and governmental systems that make it possible for Aboriginal domestic violence and abuse to continue; to develop a comprehensive framework for intervention that addresses the root causes of domestic violence and abuse in Aboriginal communities, and identifies an integrated set of strategies for significantly reducing the currently horrendous levels of domestic violence and abuse | **Despite some progress in the field there are still large gaps between what is actually occurring in relation to domestic violence in Aboriginal communities** and the capacity of these same communities and of the agencies that work with them to systematically and effectively address the problem. **Gaps are due to a lack of understanding of the complexity of domestic violence and abuse in Aboriginal communities as a social phenomenon and a set of strategies to effectively reduce violence.** |
| Bennett, L., 2001. *Controversies and recent studies of batterer intervention program effectiveness*. VAWnet: National Resource Center on Domestic Violence. | A review of **batterer intervention programs to discern effectiveness of** same; different types of evaluations-–non-experimental, quasi-experimental, and experimental evaluations--are described and the results of four previous experimental evaluations of batterer intervention programs are enumerated. Challenges include the problems of how to measure victim safety and how to address co-occurring issues, such as substance abuse, unemployment, and mental disorders that may be aggravating the abuse | **BIP’s have a small but significant effect on offenders. Factors influencing success are man's stake in conformity (education, employment, relationship commitment, community bonding), mental status (the effects of personality disorder, mental disorder, substance abuse disorder), or cultural congruity (the more** group facilitators share culture and language with the participants, the greater the stake in the group)Ongoing assessment and evaluation are essential |
| Bopp, J. and Bopp, M., 1997. *At the Time of Disclosure: A Manual for Front-line Community Workers Dealing with Sexual Abuse Disclosures in Aboriginal Communities*. Aboriginal Corrections Policy Unit, Solicitor General Canada. | Manual developed to **assist front-line community workers, especially those in aboriginal communities, deal more effectively with issues around sexual and other abuse disclosures.** | Front-line professionals (social workers, health and alcohol workers, political leaders, etc.) are caught between the pressures of two worlds — the aboriginal way of seeing the problem and the dominant society’s way of understanding and dealing with this issue. This pressure can be worsened by the demands of the **legal and bureaucratic systems, which sometimes threaten to go after community workers who don’t conform to the dominant society** approach. |
| Jaffer, Mobina (Chair) (1992). Is anyone listening? Report of the British Columbia **Task Force on Family Violence**. Victoria, BC: Minister of Women’s Equality.  Sinha, Mair (2013) Prevalence and Severity of Violence Against Women,  Minister of Industry, **Statistics** Canada, Ottawa | 29% of women in Canada who had ever been married or lived in a common-law relationship reported being physically or sexually assaulted by a marital partner at least once during the relationship.  **British Columbia has the highest reported rates of violence against women of any province in Canada.**  Data from the Homicide Survey indicate that Aboriginal women were disproportionally represented as homicide victims. Similarly, **victimization data indicate that Aboriginal women have higher rates of self-reported spousal and non-spousal violence.** | There are enormous challenges to incorporating the perspectives of women who experience violence (Lawrence, 1996; Winters, 1992). Women in violent relationships are rarely in a position to participate and those who have left such relationships are often struggling just to survive. While many working in anti-violence have experienced violence themselves, the dominant perspectives are professionalized, and there are a wide variety of views. |
| Stoker, S.L., 1998. An examination of the **ability of mainstream institutions to meet the needs of Aboriginal victims** of domestic violence. | **Past theory and research** in the area of domestic violence in Aboriginal communities have **argued that mainstream institutions are not meeting the needs of Aboriginal women** because their mandates do not incorporate Aboriginal traditions and philosophies. An exploratory, descriptive investigation was conducted which focusses on the **utilization of mainstream services by Aboriginal women, the satisfaction with mainstream services by its users and the ability of mainstream services to meet the needs of Aboriginal women.** | The results of this research failed to support the theoretical argument that mainstream institutions are not meeting the needs of Aboriginal victims of domestic violence because they are not based upon traditional Aboriginal philosophies and customs. |
|  |  |  |
| Smith, D., Varcoe, C. and Edwards, N., 2005. Turning around the intergenerational impact of residential schools on Aboriginal people: Implications for health policy and practice. *CJNR (Canadian Journal of Nursing Research)*, *37*(4), pp.38-60. | Two thirds of that last generation to attend residential schools has not survived. It is no coincidence that so many fell victim to violence, accidents, addictions and suicide. Today the children and grandchildren of those who went to residential schools also live with the same legacy of broken families, broken culture and broken spirit. (Chief Councillor Charlie Cootes, cited in Royal Commission on Aboriginal Peoples [RCAP], 1996, p. 22) | themes in the participants’ stories serve as a prescription for action, on the part of both Aboriginal and non-Aboriginal people, with regard to relationships, programs, and policies to bring the culture back by acknowledging the profound effects of IGIRS, emphasizing healing, and focusing on strength and capacity. Such a perspective will enable health policy, organizations, and providers to work in closer harmony with Aboriginal people to achieve their vision, instead of reinforcing the colonizing relations that are a legacy of the past and a feature of everyday practice. Further research is needed to explicate competency development processes that will build partnerships between Aboriginal organizations and communities and to identify implications and actions for the nursing education and practice organizations that oversee our professional responsibilities to the public. |
|  |  |  |
| Quinn, A., 2007. Reﬂections on Intergenerational Trauma: Healing as a Critical Intervention. *First peoples child & family review*, *3*(4), pp.72-82. | Residential schools and the trauma that was experienced has been described as a “de-feathering process”, stripping Native Peoples of their knowledge, spirituality, physical and emotional well-being, and most sadly, has led to the loss of community (Locust, 2000 | Recommendations: Encourage and support further research in collaboration with First Peoples, for First Peoples, utilizing a combination of Western and First Peoples’ methodologies.  Provide funds to allow for the development of traditional healing awareness, healing programs, and further research in best healing practices. |
| Varcoe, C. and Dick, S., 2008. The intersecting risks of violence and HIV for rural Aboriginal women in a neo-colonial Canadian context. *Journal de la santé autochtone*. | Neo-colonial and racist context of Canadian society creates particular challenges for Aboriginal women.  Aboriginal women face more structural inequities; therefore they are at greater risk of experiencing violence, poverty and poor housing. Consequences of systemic racism have included poverty, disconnection from family and community, and feelings of despair | Understanding how the intersecting dynamics of gender, rural living, poverty, racism, and colonialism create risk for Aboriginal women provides a basis for developing policies that aim to strengthen the well-being of women, |
| Brach, C. and Fraserirector, I., 2000. Can cultural competency reduce racial and ethnic health disparities? A review and conceptual model. *Medical Care Research and Review*, *57*(4 suppl), pp.181-217. | Develops a conceptual model of cultural competency’s potential to reduce racial and ethnic health disparities | The authors identify nine major cultural competency techniques: interpreter services, recruitment and retention policies, training, coordinating with traditional healers, use of community health workers, culturally competent health promotion, including family/community members, immersion into another culture, and administrative and organizational accommodations |
